# Why psychiatric bed capacity varies widely: Strategic questions on global mental health

**DOI:** 10.1371/journal.pmed.1004761

**Published:** 2025-10-10

**Authors:** Akihiro Seita

**Affiliations:** UNRWA (United Nations Relief and Works Agency for Palestine Refugees), Amman, Jordan

## Abstract

A recent *PLOS Medicine* study reveals the wide variation in psychiatric bed numbers across the US. Globally, capacity differs 80-fold among OECD countries, reflecting history, policy, and care models. Without global standards and access, the efficiency and quality of psychiatric care remain uneven.

Access to mental healthcare is an urgent public health challenge. In the United States, the adequacy and distribution of inpatient psychiatric beds have become a particular concern, as they remain essential for treating people in acute psychiatric crisis. To address these questions, a recent *PLOS Medicine* study [1] presents a detailed analysis of trends in the US, reporting a national average of 0.35 psychiatric beds per 1,000 population and substantial variation between states and districts. States with the lowest *per capita* capacity have fewer than half as many beds as those with the highest capacity. The authors interpret this variation as a potential indicator of unequal access to inpatient psychiatric care. While this framing is important, such variation may also reflect differences in how mental healthcare is organized, financed, and delivered across jurisdictions.

The findings of this study mirror patterns seen internationally. Among the 37 high-income members of the Organization for Economic Co-operation and Development (OECD), psychiatric bed numbers differ by more than 80-fold (Fig 1 OECD countries at large on the left, and OECD countries in Europe on the right) [2]. OECD data from 2022 show Japan with 2.58 psychiatric beds per 1,000 population, Italy with just 0.08, and the United States at 0.35. The median among OECD countries is around 0.64 beds per 1,000 [2]. These disparities cannot be explained by the burden of mental health conditions or care needs alone [3]. Instead, they reflect a combination of historical approaches to mental healthcare, national policy choices, financing structures, clinical cultures, and the organization of national health systems [3,4].

**Fig 1:**
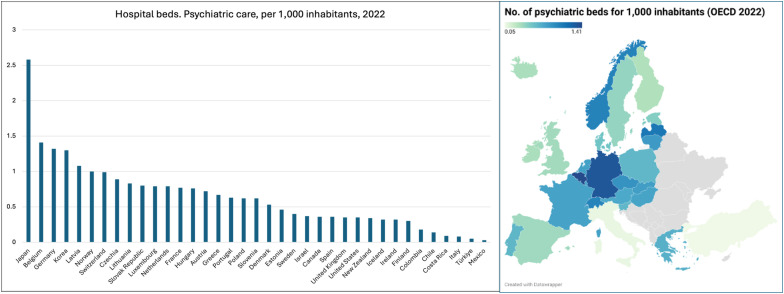
International variation in the number of psychiatric beds per 1,000 inhabitants (2022). **(Left)** Psychiatric bed numbers across OECD countries. **(Right)** A map visualizing bed capacity variations across Europe. Graph source: https://www.datawrapper.de/_/Ua4a0/?v=3. Data available from the OECD data explorer: https://data-explorer.oecd.org/vis?pg=0&bp=true&snb=6&df[ds]=dsDisseminateFinalDMZ&df[id]=DSD_HEALTH_REAC_HOSP%40DF_BEDS_FUNC&df[ag]=OECD.ELS.HD&df[vs]=1.0&dq=.10P3HB&_T._T.&pd=%2C&to[TIME_PERIOD]=false&vw=tb&lc=en. Map base layer from Natural Earth (public domain): https://www.naturalearthdata.com/about/terms-of-use/. Visualization created with Datawrapper (https://www.datawrapper.de).

In countries with high numbers of psychiatric beds, such as Japan, capacity may partly reflect an aging population and the prevalence of dementia or other conditions requiring long-term care [5]. Yet demographic and medical factors alone cannot explain the magnitude of the difference [3]. In many OECD countries, current bed numbers are the result of decades of deinstitutionalization policies [6], shifts toward community- and primary care-based mental health services [4], and changes in how acute psychiatric crises are managed [4]. Where well-resourced primary healthcare services exist, lower inpatient bed numbers may be offset by timely access to outpatient care, crisis intervention, and supported care homes. Conversely, where such systems are underdeveloped, fewer inpatient beds can lead to bottlenecks, inappropriate admissions to general hospitals, or limited access to care altogether [4].

The contrast is even starker beyond high-income countries. In many low- and middle-income countries, psychiatric bed capacity is often below 0.1 per 1,000 population [3], far lower than the OECD median. This scarcity reflects constrained health budgets, limited infrastructure, and shortages of trained mental health professionals. Psychiatric inpatient services are frequently concentrated in a few large urban hospitals, creating significant geographic barriers for much of the population. Where care is provided in private hospitals, costs can become a major barrier for people living with mental health conditions [3]. Admissions are typically reserved for individuals with the most acute conditions, and length of stay is often determined by bed turnover pressures rather than clinical need. For many, no formal mental healthcare is available at all, leaving families and informal community networks to manage complex psychiatric conditions without professional support [3].

A recent international Delphi study proposed a minimum of 30–60 psychiatric beds per 100,000 population, seeking to balance risks of under- and over-provision. While this study is highly appreciated, the range is based on expert judgment rather than burden-of-disease estimates or outcome-based evidence, and remains broad, context-dependent, and not universally adopted, underscoring the continuing absence of a clear global benchmark [7]. These global patterns still point to a striking gap: There is no widely accepted international benchmark or norm for psychiatric bed capacity [4]. In the absence of such a reference point, countries set their own targets based on historical precedent, policy priorities, specialist preferences, and available resources rather than on agreed measures of population need. The lack of standards also leaves key operational questions unresolved: How many individuals, and with which mental health conditions, should be managed entirely in outpatient settings? What constitutes appropriate admission criteria? How long should inpatient stays last? And what is the optimal balance between acute psychiatric units and longer-term rehabilitation beds?

The absence of an agreed benchmark or norm for psychiatric bed capacity may contribute to inequities, inefficiencies, and fragmented mental healthcare. Countries with very low bed numbers risk denying people with mental health conditions a rights-based approach to care, leading to untreated or deteriorating medical conditions and adverse social outcomes such as isolation, restraint in the home, reliance on traditional or religious healing sites, warehousing in social care institutions, or even imprisonment [3]. At the other extreme, very high bed numbers may signal over-reliance on institutional care, potentially diverting resources from primary healthcare-based services and perpetuating stigma toward mental health conditions. Without a shared standard, it is difficult to assess whether any country’s psychiatric bed capacity is proportionate, efficient, or effective.

Any attempt to standardize psychiatric bed numbers must be grounded in a clear, internationally agreed mental healthcare strategy. The WHO Comprehensive Mental Health Action Plan (2013–2030) provides overall strategic direction [8], emphasizing a shift away from large psychiatric institutions toward community- and general hospital-based services. Any development of global benchmarks for psychiatric bed numbers must also be aligned with rights-based, non-coercive care principles, as emphasized in the UN Convention on the Rights of Persons with Disabilities, together with people with lived experience to ensure acceptability and safety. Expanding such frameworks to include guidance on inpatient care models and capacity could be a critical step toward reducing the extreme disparities in psychiatric bed availability worldwide.

Developing such a global strategy will be challenging. The wide variation in hospital bed numbers is just the visible tip of a much larger divergence in mental healthcare systems, spanning funding models, workforce capacity, clinical cultures, and the balance between inpatient and primary healthcare-based care [3,4]. Aligning these diverse approaches will require long-term commitment, political will, and sustained international collaboration [9]. Nevertheless, the findings of Lindenfeld and colleagues [1] make clear that moving toward some degree of standardization is essential to ensure fair, equitable, and universal access to mental healthcare.

## Abbreviation:

OECD: Organization for Economic Co-operation and Development
